# Common genetic variability in *ESR1* and *EGF* in relation to endometrial cancer risk and survival

**DOI:** 10.1038/sj.bjc.6604984

**Published:** 2009-03-24

**Authors:** K Einarsdóttir, H Darabi, K Czene, Y Li, Y L Low, Y Q Li, C Bonnard, S Wedrén, E T Liu, P Hall, J Liu, K Humphreys

**Affiliations:** 1 Centre for Health Services Research, School of Population Health, University of Western Australia, Crawley, 6009 Perth, Western Australia, Australia; 2 Department of Medical Epidemiology and Biostatistics, Karolinska Institutet, 171 77 Stockholm, Sweden; 3 Human Genetics, Genome Institute of Singapore, Singapore 138672, Singapore; 4 Singapore Institute for Clinical Sciences, Brenner Centre for Molecular Medicine, Singapore 117609, Singapore; 5 Institute for Environmental Medicine, Karolinska Institutet, 171 77 Stockholm, Sweden; 6 Cancer Biology, Genome Institute of Singapore, Singapore 138672, Singapore

**Keywords:** ESR1, EGF, polymorphism, endometrial cancer, survival

## Abstract

We investigated common genetic variation in the entire *ESR1* and *EGF* genes in relation to endometrial cancer risk, myometrial invasion and endometrial cancer survival. We genotyped a dense set of single-nucleotide polymorphisms (SNPs) in both genes and selected haplotype tagging SNPs (tagSNPs). The tagSNPs were genotyped in 713 Swedish endometrial cancer cases and 1567 population controls and the results incorporated into logistic regression and Cox proportional hazards models. We found five adjacent tagSNPs covering a region of 15 kb at the 5′ end of *ESR1* that decreased the endometrial cancer risk. The *ESR1* variants did not, however, seem to affect myometrial invasion or endometrial cancer survival. For the *EGF* gene, no association emerged between common genetic variants and endometrial cancer risk or myometrial invasion, but we found a five-tagSNP region that covered 51 kb at the 5′ end of the gene where all five tagSNPs seemed to decrease the risk of dying from endometrial cancer. One of the five tagSNPs in this region was in strong linkage disequilibrium (LD) with the untranslated A61G (rs4444903) *EGF* variant, earlier shown to be associated with risk for other forms of cancer.

Endogenous and exogenous oestrogen exposure is a central factor in the development and progression of endometrial cancer (Persson, 2000), which is the most common gynaecological cancer in the industrialised world. Oestrogen receptor *α* (ESR1) is the main mediator of oestrogen effect in epithelium, including the endometrium (Matsuzaki *et al*, 1999). The gene coding for the ESR1 protein could thus play a role in susceptibility and prognosis of endometrial cancer. Another potential role player in endometrial cancer aetiology is the epidermal growth factor (EGF). This protein can activate ESR1 and acts as a potent mitogen for epithelial cells (Nelson *et al*, 1991; Ignar-Trowbridge *et al*, 1992; Curtis *et al*, 1996; Dickson and Lippman, 1998; Marquez *et al*, 2001). We intended to study common variation in both the *ESR1* (MIM 133430) and *EGF* (MIM 131530) genes in relation to endometrial cancer risk, myometrial invasion and endometrial cancer survival. The common variation in the *EGF* gene has never before been investigated with regard to endometrial cancer susceptibility, but a few studies have been published regarding *ESR1* and endometrial cancer risk (Weiderpass *et al*, 2000; Sasaki *et al*, 2002; Iwamoto *et al*, 2003). The earlier investigators have, however, concentrated only on few single-nucleotide polymorphisms (SNPs) in the gene. We aimed towards capturing the entire common variation in the *ESR1* and *EGF* genes by genotyping a dense set of markers in 92 Swedish controls and then selecting haplotype tagging SNPs (tagSNPs) that were genotyped in 713 Swedish endometrial cancer cases and 1567 Swedish controls. We assessed the association of the tagSNPs with endometrial cancer risk, myometrial invasion and endometrial cancer survival using logistic regression and Cox regression models.

## Materials and methods

### Study population and DNA extraction

Details of the population selection process for this study have been published earlier (Einarsdottir *et al*, 2007). In brief, 68% (719) of all endometrial cases among women 50–74 years of age identified through the nation-wide cancer registries in Sweden between 1994 and 1995 agreed to participate in this study. During that period, 64% (1574) of the age-frequency matched controls selected from the Swedish Registry of Total Population agreed to participate. Only women with an intact uterus were considered eligible as controls. All participants provided detailed questionnaire information, and histological specimens for all the endometrial cases were reviewed and re-classified by the study pathologist.

Following informed consent, 603 cases and 1574 controls donated whole blood for DNA extraction. For deceased cases and those cases that declined to donate blood but consented to our use of tissue, we collected archived paraffin-embedded, non-cancerous tissue samples (*n*=116). We extracted DNA from 4 ml of whole blood using the QIAamp DNA Blood Maxi Kit (Qiagen, Solna, Sweden) according to the manufacturer′s instructions. From non-malignant cells in paraffin-embedded tissue, we extracted DNA using a standard phenol/chloroform/isoamyl alcohol protocol (Isola *et al*, 1994). We successfully isolated DNA from 600 (blood) and 116 (tissue) endometrial cancer patients and 1567 (blood) controls. We randomly selected 92 controls out of the 1567 controls to be used for linkage disequilibrium (LD) characterisation and haplotype reconstruction of the *ESR1* and *EGF* genes.

This study was approved by the Institutional Review Boards in Sweden and at the National University of Singapore.

### SNP markers and genotyping

The *ESR1* gene covers 295.7 kb of genomic sequence on chromosome 6, and *EGF* spans 99.4 kb on chromosome 4. We selected SNPs in the *ESR1* and *EGF* genes and their 20-kb flanking sequences from dbSNP (build 124) and Celera databases, aiming for an initial marker density of at least one SNP per 5 kb. SNPs were genotyped at the Genome Institute of Singapore using the Sequenom primer extension-based assay (Sequenom, San Diego, CA, USA) and the BeadArray system from Illumina (San Diego, CA, USA) following the manufacturers' instructions. All genotyping plates included positive and negative controls, DNA samples were randomly assigned to the plates, and all genotyping results were generated and checked by laboratory staff unaware of case–control status. Only SNPs where >85% of the samples gave a genotype call were analysed further. As quality control, we genotyped 200 randomly selected SNPs in the 92 control samples using both the Sequenom system and the BeadArray system. The genotype concordance was >99.5%, suggesting high genotyping accuracy and high concordance between the two platforms.

### LD characterisation and TagSNP selection

We successfully genotyped 228 SNPs in the *ESR1* gene and 104 SNPs in the *EGF* gene in the 92 controls. The SNP names, physical positions, minor allele frequencies (MAF) and the Hardy–Weinberg equilibrium (HWE) *P*-values have been published earlier as Supplementary Tables 1 and 2 in Einarsdóttir *et al* (2008). We thereafter identified regions of LD and selected ‘tagging’ SNPs (tagSNPs). We produced LD plots of the *D′* and *R*^*2*^ values for *ESR1* and *EGF* using the *LDheatmap* function in the statistical software R (Team, 2005). The plots have been published earlier as Supplementary Figures 1 and 2 in Einarsdóttir *et al* (2008). We reconstructed haplotypes for the genes using the PLEM algorithm (Qin *et al*, 2002) implemented in the *tagSNPs* program (Stram *et al*, 2003) and selected tagSNPs based on the *R*^*2*^ coefficient, which quantifies how well the tagSNP haplotypes predict the SNPs or the number of copies of haplotypes an individual carries. We chose tagSNPs so that common SNP genotypes (minor allele frequency ⩾0.03) and common haplotypes (frequency ⩾0.03) were predicted with *R*^*2*^⩾0.8 (Gabriel *et al*, 2002). The well-studied *Pvu*II (rs2234693), *Xba*I (rs9340799), codon 243 (rs4986934) and codon 325 (rs1801132) variants in *ESR1* had been genotyped earlier in our study subjects (Wedrén *et al*, 2008), in both cases and controls, and were therefore ‘forced’ into the selection of tagSNPs. In order to evaluate our tagSNPs' performance in capturing unobserved SNPs within the genes and to assess whether we needed a denser set of markers, we carried out an SNP-dropping analysis (Weale *et al*, 2003; Iles, 2006). In brief, each of the genotyped SNPs was dropped in turn and tagSNPs were selected from the remaining SNPs so that their haplotypes predicted the remaining SNPs with an *R*^*2*^ value of 0.85. We then estimated how well the tagSNP haplotypes of the remaining SNPs predicted the dropped SNP, an evaluation that can provide an unbiased and accurate estimate of tagSNP performance (Weale *et al*, 2003; Iles, 2006).

### Endometrial tumour characteristics and follow-up

We retrieved information for the endometrial cancer cases on date and cause of death until 31 December 2004 from the Swedish Causes of Death Registry and on date of emigration from the Swedish National Population Registry. Follow-up time began at date of diagnosis and ended on 31 December 2004, or at the date of death or emigration, whichever came first.

Endometroid endometrial carcinomas constituted the majority of the cancers. The endometroid tumours were classified according to cell differentiation: Grade I (well-differentiated carcinomas, maximum 5% solid areas); Grade II (moderately differentiated, 6–50% solid areas); and Grade III (poorly differentiated or undifferentiated, more than 50% solid areas). Myometrial invasion was classified as present (at least 50% of the myometrial thickness or through the serosa) or absent (none or <50% of the myometrial thickness).

### Statistical analyses

We applied unconditional logistic regression models for assessing the association between *ESR1* and *EGF* tagSNPs and risk of endometrial cancer (case–control analysis) or myometrial invasion (case-only analysis). Adjusting for age (in 5-year age groups) did not affect our results. We estimated the hazard ratio (HR) of death due to endometrial cancer in relation to the genes' tagSNP using Cox proportional hazards models. The tagSNPs were included as covariates in the models either one at a time or in groups of five (codominant main effects only). The latter method was used for detection of association with haplotypes and is referred to as the ‘sliding window’ analysis in the main text. Although it does not require resolution of gametic phase, tests based on such models can be powerful within regions of strong LD (Clayton *et al*, 2004). Likelihood ratio tests were used to generate *P*-values for comparing models with or without covariates.

Confounding has been defined as the presence of a common cause to the exposure and the outcome (Hernan *et al*, 2002). We believe that lifestyle and reproductive endometrial cancer risk factors are unlikely to cause genetic variation and we thus did not adjust for them in the analyses.

We made adjustments to our test results to account for multiplicity. We did so for each outcome (risk, myometrial invasion, and survival) separately. We used a permutation-based approach that controls for the family-wise error rate (probability of rejecting one or more true null hypotheses of no association). Analyses were carried out using the statistical software R (Team, 2005) and the SAS system (release 9.1, SAS Institute Inc., Cary, NC, USA).

## Results

### Characteristics of participants

Table 1 shows selected characteristics of the study participants. Statistically significant differences between cases and controls reflected established associations.

Those cases who participated in our study through tissue sample donation were on average 2.1 years older than the cases who donated a blood sample (*P*=0.002). The former group was also more likely to have poorly differentiated (grade 3) tumours (*P*=0.08). As no significant differences in genotype frequencies within Grade 1, Grade 2, and Grade 3 were evident between the two groups of cases (data not shown), this difference is unlikely to be a cause for concern.

### Genotyping, LD pattern, and coverage

We selected a dense set of markers in the *ESR1* and *EGF* genes for genotyping in 92 randomly selected controls. We successfully genotyped 228 SNPs in the *ESR1* gene and 104 SNPs in the *EGF* gene in the 92 controls. The SNP names, physical positions, MAF, and HWE *P*-values have been published earlier as Supplementary Tables 1 and 2 in Einarsdóttir *et al* (2008). Summary statistics on genotyping results and SNP coverage for the *ESR1* and *EGF* genes are shown in Table 2. Out of the SNPs successfully genotyped in the 92 controls, those SNPs that conformed to HWE and that were at least 3% in MAF were included in our study (Table 2). We thus included 157 SNPs in *ESR1* and 54 SNPs in *EGF* in our study for LD mapping and tagSNP selection. The LD plots from the SNPs included in our study have been published earlier as Supplementary Figures 1 and 2 in Einarsdóttir *et al* (2008).

Using the SNP-dropping method (Weale *et al*, 2003), we assessed the ability of the selected tagSNPs to capture variation in the SNPs we did not genotype in our study. We found that the tagSNPs could efficiently capture non-genotyped SNPs in the genes (Table 2).

### Association analyses

We selected 52 tagSNPs in *ESR1* and 15 tagSNPs in *EGF* that could predict the SNPs included in our study and their haplotypes with an *R*^*2*^ of at least 0.8. The tagSNPs were genotyped in all cases and controls (see Supplementary Table 1 for numbers as well as MAF and HWE calculations), but seven tagSNPs in *ESR1* and one tagSNP in *EGF* could not be genotyped in the cases who participated through tissue sample donation.

### ESR1

**Figure 1:**
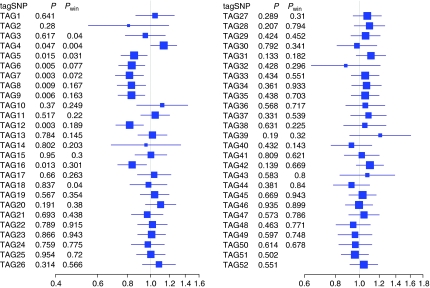
Association of the 52 tagSNPs in *ESR1* with endometrial cancer risk. Squares and horizontal lines represent odds ratios (change in risk with each addition of the rare allele) and their confidence intervals. Sizes of the squares reflect the minor allele frequencies. *P*=*P*-value for an association of each tagSNP with endometrial cancer risk. *P*_win_=*P*-value from a model including a window of five tagSNPs (the *P*-value aligns with the middle tagSNP of each window) for an association with endometrial cancer risk.

*P*-values and odds ratios (ORs) for association of each tagSNP in *ESR1* with endometrial cancer risk, myometrial invasion and endometrial cancer survival are presented in Figure 1, Supplementary Figure 1, and Supplementary Figure 2, respectively. The figures also show *P*-values (*P*_win_) generated from a sliding window approach where five adjacent tagSNPs were analysed together in a regression model (without resolution of gametic phase).

The single tagSNP analysis indicated a 15-kb region of five tagSNPs – including TAG5 (rs3853250) to TAG9 (rs1709181) – that were all associated with endometrial cancer risk (Figure 1), but that did not withstand multiple testing correction. The sliding window analysis including TAGs 5–9 gave a *P*-value of 0.072 (Figure 1). This region – which covered intron 1 and intron 2 at the 5′ end of *ESR1* – included the *Pvu*II (TAG6, OR 0.83, 95% CI 0.74–0.95) and *Xba*I (TAG7, OR 0.82, 95% CI 0.71–0.94) variants. We show individual genotype associations for the five tagSNPs in Table 3.

We reconstructed haplotypes from TAGs 5–9 and found that five common (>0.03) haplotypes accounted for 95% of the chromosomes. We included expected ‘dosages’ of the five common haplotypes and the rare haplotypes (combined into a single group) as covariates in a logistic regression model with the most common haplotype as reference (Stram *et al*, 2003). We found a haplotype (haplotype 2) that carried all the five minor alleles of TAGs 5–9 was associated with endometrial cancer risk (*P*=0.0026), compared with haplotype 1, which carried none of the minor alleles (Table 4). This association did not carry over to the global test (*P*=0.067).

The strongest signal in relation to endometrial cancer risk (Figure 1) came from TAG12 (rs1033182, OR 0.82, 95% CI 0.72–0.94, *P*=0.003) – situated 20 kb from TAG9 – but this again did not withstand adjustment for multiple testing (*P*=0.154). When we carried out the single tagSNP analyses within groups defined by duration of menopausal oestrogen only use or family history, we found that the protective effect of TAG12 on endometrial cancer risk was to a large extent confined to never users of menopausal oestrogen. Yet, the *P*-value for interaction did not suggest that the effect of TAG12 depended on oestrogen only use (*P*=0.18).

Neither the single tagSNP analysis nor the window analysis showed an association of common genetic variation in the *ESR1* gene with myometrial invasion (Supplementary Figure 1) or endometrial cancer survival (Supplementary Figure 2).

### EGF

**Figure 2:**
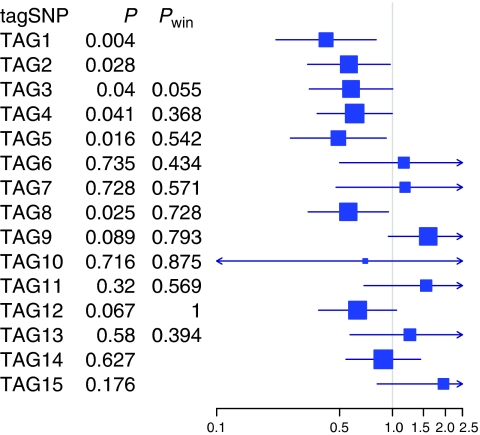
Association of the 15 tagSNPs in *EGF* with endometrial cancer survival. Squares and horizontal lines represent hazard ratios (change in risk with each addition of the rare allele) and their confidence intervals. Sizes of the squares reflect the minor allele frequencies. *P*=*P*-value for an association of each tagSNP with endometrial cancer survival. *P*_win_=*P*-value from a model including a window of five tagSNPs (the *P*-value aligns with the middle tagSNP of each window) for an association with endometrial cancer survival.

Our data did not indicate that common genetic variants in the *EGF* gene are associated with endometrial cancer risk (Supplementary Figure 3) or myometrial invasion (Supplementary Figure 4). With regard to endometrial cancer survival, the single tagSNP analysis signified a 51-kb 5′ region of five tagSNPs in *EGF* – including TAG1 (rs718768, HR 0.42, 95% CI 0.22–0.81, *P*=0.0038) to TAG5 (rs1024600, HR 0.49, 95% CI 0.26–0.92, *P*=0.016) – that were associated with the risk of dying from endometrial cancer (Figure 2). The association signals were, however, rendered not significant after multiple testing adjustment. The sliding window analysis including TAGs 1–5 gave a *P*-value of 0.055 for endometrial cancer survival (Figure 2). Table 3 shows individual genotype associations for the five tagSNPs in *EGF*.

In this region of *EGF*, 10 kb downstream of TAG1 and 2 kb upstream of TAG2 (rs881878), lies the untranslated A61G (rs4444903) polymorphism. This polymorphism was genotyped in our 92 Swedish controls and was in LD with TAG2 (*R*^*2*^=0.84). TAG2 was associated with the risk of dying from endometrial cancer with a HR of 0.57 (95% CI 0.33–0.97, *P*=0.028).

When we reconstructed haplotypes from TAGs 1–5 in *EGF*, we found that four common haplotypes (>0.03) accounted for 95% of the chromosomes in the cases. We constructed a Cox proportional hazards model including the expected ‘dosages’ of the common haplotypes and rare haplotypes (combined into a single group) with the most common haplotype as reference (Table 4). A haplotype (haplotype 2) carrying all rare alleles of the five tagSNPs decreased the risk of dying from endometrial cancer (*P*=0.006, HR 0.33, 95% CI 0.15–0.73) compared with a haplotype carrying none of the rare alleles. This association did not, however, carry over to the global test (*P*=0.10).

## Discussion

Using a comprehensive tagging approach of common variation in the *ESR1* and *EGF* genes, we assessed whether common variants in the genes affected endometrial cancer risk, myometrial invasion, or endometrial cancer survival. The *ESR1* gene did not seem to significantly affect myometrial invasion or endometrial cancer survival. However, a 5′ region of five tagSNPs in *ESR1* decreased endometrial cancer risk in our dataset. For the *EGF* gene, no association emerged between common variants in the gene and endometrial cancer risk or myometrial invasion, but we found a five tagSNP region at the 5′ end of the gene where all five tagSNPs seemed to decrease the risk of dying from endometrial cancer. One of the five tagSNPs in this region was in strong LD with the A61G (rs4444903) *EGF* variant. None of the association signals in *ESR1* or *EGF* withstood multiple testing correction.

Most earlier genetic association studies of the *ESR1* gene in relation to endometrial cancer risk have focused on only a few common genetic polymorphisms. Among the most commonly studied variants are the *Pvu*II (TAG6, rs2234693), *Xba*I (TAG7, rs9340799), codon 243 (TAG14, rs4986934) and codon 325 (TAG21, rs1801132) variants. Two groups that have explored both the *Pvu*II and *Xba*I variants in relation to endometrial cancer risk (Weiderpass *et al*, 2000; Iwamoto *et al*, 2003) found that the variants decreased the risk of the disease. However, a group that investigated the *Pvu*II, codon 243 and codon 325 variants found no association with endometrial cancer risk (Sasaki *et al*, 2002). In our earlier study of the *ESR1* gene, we investigated the *Pvu*II, *Xba*I, codon 243, codon 325 variants, and a 5′ promoter microsatellite (rs2234670), and found the *Pvu*II and *Xba*I variants as well as the microsatellite to be associated with endometrial cancer risk (Wedrén *et al*, 2008). In the current study, we went on to explore the gene in greater detail and found a 5′ region of five tagSNPs in the *ESR1* gene – including the *Pvu*II (TAG6, rs2234693) and *Xba*I (TAG7, rs9340799) variants – that decreased endometrial cancer risk. It is interesting that, in our earlier publication of the *ESR1* gene in relation to breast cancer risk and survival, the same region showed a tendency towards a decreased risk of breast cancer (Einarsdóttir *et al*, 2008). Nevertheless, it is unlikely that any of the SNPs genotyped within this region affect ESR1 protein structure as none of them were located in exons. It is still a possibility, however, that the SNPs themselves or one or more SNPs in LD with any of them affect the regulation of ESR1 protein expression. In fact, the *Pvu*II variant has been suggested to produce a functional binding site for the transcription factor B-myb (Herrington *et al*, 2002).

This is the first study to explore the *EGF* gene in relation to endometrial cancer risk and survival. We found five tagSNPs – one upstream of the gene and the others in the 5′ introns – that were associated with the risk of dying from endometrial cancer. This five-tagSNP region encompassed the 5′ untranslated A61G (rs4444903) polymorphism, which was in high LD with TAG2, one of the five tagSNPs. The A61G variant has been found to affect the risk of malignant melanoma (Shahbazi *et al*, 2002), hepatocellular carcinoma in patients with cirrhosis (Tanabe *et al*, 2008), gastric cancer (Hamai *et al*, 2005; Jin *et al*, 2007), and glioma (Costa *et al*, 2007). It has also been suggested to affect malignant melanoma survival (Okamoto *et al*, 2006) and oesophageal cancer survival (Jain *et al*, 2007). Furthermore, the 61^*^G allele has been found to have a significantly more active promoter (Vauleon *et al*, 2007) and produce more EGF protein than the 61^*^A allele (Shahbazi *et al*, 2002; Tanabe *et al*, 2008).

Our study was a population-based case–control study with a well-defined study base. All participants were Caucasian and born in Sweden between 1919 and 1944, at a time when foreign immigration to Sweden was still rare (Statistics Sweden, 2004), which means population stratification is of limited concern in our study. Cases were ascertained from the nation-wide Swedish Cancer Registries, that contain virtually complete data on incident cancers in Sweden (The Swedish National Board of Health and Welfare, 2006). Information on date and cause of death of the cases was obtained from the Causes of Death Registry in Sweden, which has been found to be highly reliable (Nystrom *et al*, 1995). It is therefore likely that there is little misclassification of the outcome. Differential misclassification of the exposure is also unlikely to have accounted for our results. Genotyping was carried out using genotyping methods with low error rates, all genotyping plates included positive and negative controls, DNA samples were randomly assigned to the plates, and our genotyping personnel were blinded to case–control status. We also replicated genotype calls for a subset of samples using a separate genotyping method with over 99.5% genotype concordance.

One limitation of our study was the relatively low participation rates in our study and the small number of deaths included in the survival analysis. The lack of participation may have been associated with severe disease or death. In an attempt to minimise the problem, we sought to obtain tissue samples from the deceased cases and those cases that had declined donation of a blood sample, and were able to obtain the majority of the samples requested. It is thus unlikely that the relatively low participation was related to genotype and we thus presume that the main problem with regard to lack of participation and therefore low number of deaths was decreased power in our study.

Another limitation that deserves mentioning is the fact that we were unable to genotype seven tagSNPs in *ESR1* and one tagSNP in *EGF* in the tissue samples. In the case of these eight tagSNPs being associated with severe disease, the association with risk of endometrial cancer death might have been biased towards null in our study because we could not genotype all the severe cases. None of the eight tagSNPs were actually associated with endometrial cancer survival in our study. The fact that the results were not different when we restricted our analyses to the most severe cases among those who donated blood samples indicates that the eight tagSNPs were truly not associated with severe disease.

## Figures and Tables

**Table 1:** Selected characteristics of the cases and controls participating in the present endometrial cancer study

**Entire study**	**Cases/controls (*n*)**	**Cases/controls (mean)**	***P* a**
Age (years)	713/1567	64.0/62.8	<0.0001
Age at menopause (years)	624/1508	51.0/50.1	<0.0001
Recent BMI (kg/m^2^)^b^	712/1550	27.4/25.5	<0.0001
Age at last birth (years)	610/1408	29.4/30.4	0.0006
Parity	713/1567	1.9/2.1	<0.0001
			
**Cases only**	***n* (%)**	**Endometrial cancer deaths (*n*)^c^**	**Mortality rate^d^**
Total cases	713	41	0.0064
*Endometroid tumours*	661 (92.7)	30	0.0050
Grade 1	256 (38.7)	4	0.0017
Grade 2	289 (43.7)	13	0.0048
Grade 3	116 (17.6)	13	0.0134
			
*Myometrial invasion* e			
No	399 (70.5)	12	0.0034
Yes	167 (29.5)	11	0.0081

BMI=body mass index.

a Kruskal–Wallis test of difference between cases and controls.

b One year before diagnosis.

c From the date of diagnosis until 31 December 2004.

d From the date of diagnosis until 31 December 2004 or until date of emigration, whichever came first. Calculated as endometrial cancer deaths per person-year of follow-up.

e No: No invasion or <50% of the myometrum. Yes: Invasion through ⩾50% of the myometrium or through the serosa.

**Table 2:** Genotyping results and SNP coverage in *ESR1* and *EGF*

	**ESR1**	**EGF**
Successfully genotyped SNPs^a^ (*n*)	228^b^	104^c^
Polymorphic SNPs (*n*)	184	66
Common SNPs^d^ (*n*)	165	55
SNPs deviating from HWE^e^ (*n*)	8	1
SNPs ultimately included in our study (*n*)	157	54
		
Gene size (kb)	295.7	99.4
Sequence coverage of included SNPs (kb)	335.1	145.5
Mean spacing between included SNPs (kb)	2.1	2.7
Median spacing between included SNPs (kb)	1.8	2.3
Number of tagSNPs selected	52	15
Average tagSNP prediction of common SNPs included in study (*R*^2^)^d^	0.998	0.987
		
*Coverage evaluation* f		
Average prediction of dropped SNPs (*R*^2^)	0.997	0.948
Percentage of *R*^2^ values ⩾0.7	100	96.3

EGF=epidermal growth factor; HWE=Hardy–Weinberg equilibrium; kb=kilo bases; SNPs=single-nucleotide polymorphisms.

a In 92 controls.

b Supplementary Table 1 in Einarsdóttir *et al* (2008).

c Supplementary Table 2 in Einarsdóttir *et al* (2008).

d Common was defined as minor allele frequency ⩾0.03.

e *P*<0.01.

f SNP dropping method by Weale *et al* (2003).

**Table 3:** Genotype-specific associations of five tagSNPs in *ESR1* and *EGF* with endometrial cancer risk and survival, respectively

***ESR1* – endometrial cancer risk**					
	**TAG5**	**TAG6^a^**	**TAG7^b^**	**TAG8**	**TAG9**
	**OR (95% CI)**	**OR (95% CI)**	**OR (95% CI)**	**OR (95% CI)**	**OR (95% CI)**
00	1.00 (Ref)	1.00 (Ref)	1.00 (Ref)	1.00 (Ref)	1.00 (Ref)
01	0.93 (0.75–1.14)	0.90 (0.74–1.11)	0.82 (0.68–0.99)	0.82 (0.68–0.99)	0.74 (0.60–0.90)
11	0.72 (0.55–0.93)	0.68 (0.53–0.89)	0.67 (0.49–0.92)	0.70 (0.51–0.97)	0.74 (0.57–0.97)
					
***EGF* – endometrial cancer survival**					
	**TAG1**	**TAG2**	**TAG3**	**TAG4**	**TAG5**
	**HR (95% CI)**	**HR (95% CI)**	**HR (95% CI)**	**HR (95% CI)**	**HR (95% CI)**
00	1.00 (Ref)	1.00 (Ref)	1.00 (Ref)	1.00 (Ref)	1.00 (Ref)
01	0.39 (0.18–0.86)	0.70 (0.36–1.37)	0.73 (0.37–1.44)	0.76 (0.39–1.46)	0.52 (0.24–1.12)
11	0.23 (0.03–1.65)	0.17 (0.02–1.22)	0.17 (0.02–1.29)	0.25 (0.06–1.05)	0.20 (0.03–1.50)

CI=confidence interval; EGF=epidermal growth factor; HR=hazard ratio; OR=odds ratio.

a Also named *Pvu*II (rs2234693).

b Also named *Xba*I (rs9340799).

**Table 4:** Association of haplotypes reconstructed from *ESR1* TAGs 5–9 and *EGF* TAGs 1–5 with endometrial cancer risk and survival, respectively

***ESR1* – risk**	**Haplotype proportions**			
**TAGs 5–9**	**Haplotypes**	**Cases (*n*=718^a^)**	**Controls (*n*=1575^a^)**	**OR (95% CI)**
Haplotype 1	00000	0.47	0.43	1.00 (Reference)
Haplotype 2	11111	0.26	0.29	0.79 (0.68–0.92)
Haplotype 3	11000	0.11	0.11	0.95 (0.77–1.18)
Haplotype 4	00001	0.09	0.08	0.91 (0.71–1.16)
Haplotype 5	11110	0.03	0.03	0.75 (0.50–1.13)
	Rare^b^	0.05	0.05	0.91 (0.67–1.24)
Global *P*-value^c^				0.067
				
***EGF* – survival**	**Haplotype proportions**			
**Tags 1–5**	**Haplotypes**	**Cases (*n*=703^a^)**		**HR (95% CI)**
Haplotype 1	00000	0.59		1.00 (Reference)
Haplotype 2	11111	0.23		0.33 (0.15–0.73)
Haplotype 3	01111	0.07		0.98 (0.45–2.16)
Haplotype 4	00010	0.06		0.86 (0.34–2.19)
	Rare^d^	0.05		0.70 (0.22–2.21)
Global *P*-value^c^				0.10

CI=confidence interval; EGF=epidermal growth factor; HR=hazard ratio; OR=odds ratio.

a Information on at least one of the five tagSNPs.

b 15 rare haplotypes combined. Each haplotype has frequency below 3%.

c Likelihood ratio test.

d 8 rare haplotypes combined. Each haplotype has frequency below 3%.
