# Supplementary material for: Common genetic variability in ESR1 and EGF in relation to endometrial cancer risk and survival
Source: Br J Cancer. 2009 Mar 24;100(8):1358–64. doi: 10.1038/sj.bjc.6604984 (PMC2676544; doi:10.1038/sj.bjc.6604984)
Supplement: Supplementary Information [file 6604984x1.doc]

Supplementary Table 1. Summary of the tagSNPs in *ESR1* and *EGF* that were genotyped in all endometrial cancer cases and controls.

| tagSNPs | SNP name | Number of cases/controls | MAF^a^ | HWE  *P*-value^a,b^ |  | tagSNPs | SNP name | Number of cases/controls | MAF^a^ | HWE  *P*-value^a,b^ |
| --- | --- | --- | --- | --- | --- | --- | --- | --- | --- | --- |
| **ESR1** |  |  |  |  |  | TAG35 | rs3020432 | 688/1545 | 0.38 | 0.97 |
| TAG1 | rs3853248 | 677/1505 | 0.13 | 0.90 |  | TAG36 | rs1884152 | 688/1534 | 0.14 | 0.84 |
| TAG2 | rs9371557 | 676/1483 | 0.03 | 0.18 |  | TAG37 | rs3020372 | 688/1545 | 0.17 | 0.95 |
| TAG3 | rs7775047 | 575/1530 | 0.15 | 0.69 |  | TAG38 | rs3822990 | 697/1546 | 0.11 | 0.32 |
| TAG4 | rs827421 | 690/1534 | 0.49 | 0.27 |  | TAG39 | rs2982900 | 693/1541 | 0.05 | 0.78 |
| TAG5 | rs3853250 | 689/1540 | 0.47 | 0.31 |  | TAG40 | rs3778099 | 665/1506 | 0.14 | 0.15 |
| TAG6^c^ | rs2234693 | 698/1561 | 0.47 | 0.50 |  | TAG41 | rs2228480 | 685/1532 | 0.19 | 0.73 |
| TAG7^d^ | rs9340799 | 698/1562 | 0.35 | 0.79 |  | TAG42 | rs3798577 | 690/1522 | 0.49 | 0.95 |
| TAG8 | rs4870057 | 693/1538 | 0.34 | 0.48 |  | TAG43 | rs1062577 | 680/1525 | 0.06 | 0.92 |
| TAG9 | rs1709181 | 682/1523 | 0.42 | 0.86 |  | TAG44 | rs2813543 | 519/1382 | 0.23 | 0.20 |
| TAG10 | rs1709180 | 682/1520 | 0.07 | 0.82 |  | TAG45 | rs1543403 | 687/1527 | 0.50 | 0.94 |
| TAG11 | rs1709183 | 696/1547 | 0.28 | 1.00 |  | TAG46 | rs910416 | 686/1544 | 0.45 | 0.76 |
| TAG12 | rs1033182 | 680/1509 | 0.38 | 0.45 |  | TAG47 | rs7450824 | 687/1520 | 0.27 | 0.61 |
| TAG13 | rs6557168 | 579/1523 | 0.34 | 0.72 |  | TAG48 | rs2813552 | 678/1497 | 0.21 | 0.62 |
| TAG14^e^ | rs4986934 | 701/1563 | 0.04 | 0.17 |  | TAG49 | rs2813559 | 686/1530 | 0.20 | 0.24 |
| TAG15 | rs6557170 | 693/1541 | 0.22 | 0.19 |  | TAG50 | rs11757692 | 696/1540 | 0.23 | 0.05 |
| TAG16 | rs11155820 | 692/1535 | 0.32 | 0.23 |  | TAG51 | rs2295194 | 693/1538 | 0.44 | 1.00 |
| TAG17 | rs1514347 | 520/1391 | 0.24 | 0.08 |  | TAG52 | rs9383609 | 686/1519 | 0.41 | 0.37 |
| TAG18 | rs988328 | 695/1544 | 0.15 | 0.89 |  |  |  |  |  |  |
| TAG19 | rs12154178 | 676/1526 | 0.28 | 0.56 |  | **EGF** |  |  |  |  |
| TAG20 | rs4583998 | 532/1385 | 0.29 | 0.02 |  | TAG1 | rs718768 | 696/1545 | 0.27 | 0.01 |
| TAG21^f^ | rs1801132 | 702/1563 | 0.22 | 0.96 |  | TAG2 | rs881878 | 689/1546 | 0.32 | 0.34 |
| TAG22 | rs3020314 | 588/1551 | 0.32 | 0.84 |  | TAG3 | rs3822288 | 690/1545 | 0.32 | 0.27 |
| TAG23 | rs3020377 | 700/1553 | 0.32 | 0.92 |  | TAG4 | rs2282784 | 687/1534 | 0.39 | 0.23 |
| TAG24 | rs3020317 | 672/1517 | 0.21 | 0.51 |  | TAG5 | rs1024600 | 664/1488 | 0.30 | 0.12 |
| TAG25 | rs1884051 | 688/1531 | 0.31 | 0.28 |  | TAG6 | rs7670908 | 685/1530 | 0.07 | 0.53 |
| TAG26 | rs3003925 | 678/1504 | 0.19 | 0.98 |  | TAG7 | rs9991367 | 669/1515 | 0.06 | 0.72 |
| TAG27 | rs3020318 | 695/1546 | 0.34 | 0.72 |  | TAG8 | rs7692976 | 663/1499 | 0.40 | 0.96 |
| TAG28 | rs726281 | 664/1474 | 0.26 | 0.42 |  | TAG9 | rs4698803 | 683/1531 | 0.25 | 0.10 |
| TAG29 | rs3020407 | 683/1542 | 0.32 | 0.58 |  | TAG10 | rs7653900 | 561/1485 | 0.08 | 0.65 |
| TAG30 | rs2144025 | 505/1362 | 0.18 | 0.02 |  | TAG11 | rs10021697 | 660/1476 | 0.05 | 0.21 |
| TAG31 | rs6905370 | 691/1540 | 0.29 | 0.54 |  | TAG12 | rs17320827 | 696/1536 | 0.34 | 0.49 |
| TAG32 | rs9340994 | 696/1548 | 0.04 | 0.22 |  | TAG13 | rs730598 | 695/1543 | 0.07 | 0.43 |
| TAG33 | rs3020411 | 688/1533 | 0.37 | 0.65 |  | TAG14 | rs6533489 | 672/1511 | 0.35 | 0.36 |
| TAG34 | rs926778 | 663/1493 | 0.32 | 0.85 |  | TAG15 | rs10454793 | 666/1514 | 0.04 | 0.63 |

^a^ Among all controls

^b^ Chi-square tests

^c^ Also named pvuII

^d^ Also named xbaI

^e^ Also named codon 243

^f^ Also named codon 325

MAF = Minor allele frequency, HWE = Hardy-Weinberg Equilibrium


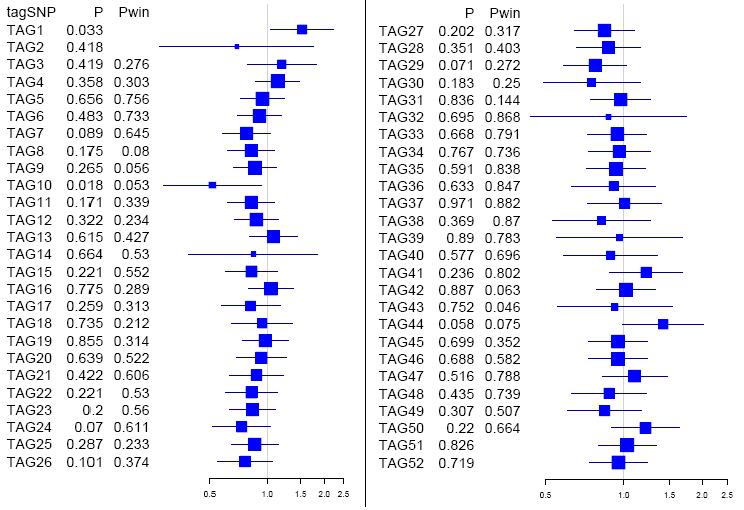


Supplementary Figure 1. Association of the 52 tagSNPs in *ESR1* with myometrial invasion (case only analysis).

Myometrial invasion was categorized into No or Yes where No equalled no invasion or invasion through <50% of the myometrum and Yes equalled invasion through ≥ 50% of the myometrum or through the serosa. Squares and horizontal lines represent odds ratios (change in risk with each addition of the rare allele) and their confidence intervals. Sizes of the squares reflect the minor allele frequencies. P = *P*-value for an association of each tagSNP with myometrial invasion. Pwin = *P*-value from a model including a window of five tagSNPs (the *P*-value aligns with the middle tagSNP of each window) for an association with myometrial invasion.


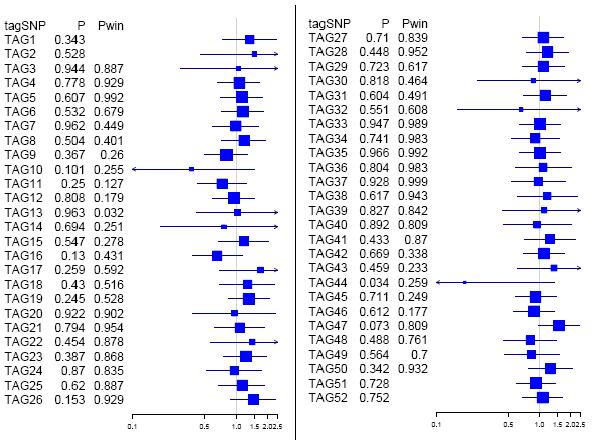


Supplementary Figure 2. Association of the 52 tagSNPs in *ESR1* with endometrial cancer survival.

Squares and horizontal lines represent hazard ratios (change in risk with each addition of the rare allele) and their confidence intervals. Sizes of the squares reflect the minor allele frequencies. P = *P*-value for an association of each tagSNP with endometrial cancer survival. Pwin = *P*-value from a model including a window of five tagSNPs (the *P*-value aligns with the middle tagSNP of each window) for an association with endometrial cancer survival.


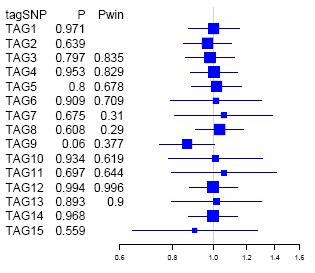


Supplementary Figure 3. Association of the 15 tagSNPs in *EGF* with endometrial cancer risk.

Squares and horizontal lines represent odds ratios (change in risk with each addition of the rare allele) and their confidence intervals. Sizes of the squares reflect the minor allele frequencies. P = *P*-value for an association of each tagSNP with endometrial cancer risk. Pwin = *P*-value from a model including a window of five tagSNPs (the *P*-value aligns with the middle tagSNP of each window) for an association with endometrial cancer risk.


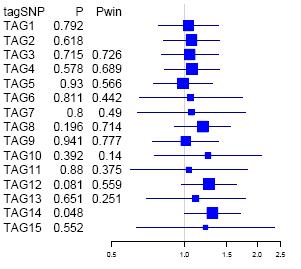


Supplementary Figure 4. Association of the 15 tagSNPs in *EGF* with myometrial invasion (case only analysis).

Myometrial invasion was categorized into No or Yes where No equalled no invasion or invasion through <50% of the myometrum and Yes equalled invasion through ≥ 50% of the myometrum or through the serosa. Squares and horizontal lines represent odds ratios (change in risk with each addition of the rare allele) and their confidence intervals. Sizes of the squares reflect the minor allele frequencies. P = *P*-value for an association of each tagSNP with myometrial invasion. Pwin = *P*-value from a model including a window of five tagSNPs (the *P*-value aligns with the middle tagSNP of each window) for an association with myometrial invasion.
